# Diagnostic imaging and pathological findings of an abdominal mesenteric granular cell tumour in a dog

**DOI:** 10.1002/vms3.543

**Published:** 2021-05-22

**Authors:** Alejandro Ororbia, Alba Sanz, Rosa Novellas, Josep Pastor, Marti Pumarola, Laura Fresno, Yvonne Espada

**Affiliations:** ^1^ Fundació Hospital Clinic Veterinari Universitat Autònoma de Barcelona Barcelona Spain; ^2^ Departamento de medicina y cirugía animal Universitat Autònoma de Barcelona Barcelona Spain; ^3^ Unit of Murine and Comparative Pathology (UPMiC) and Networking Research Center on Bioengineering, Biomaterials and Nanomedicine (CIBER‐BBN) Barcelona Spain

**Keywords:** canine, duodenum, fat, intestine, myoblastoma, veterinary

## Abstract

A 12‐year‐old mixed‐breed dog was presented for a follow‐up examination after ablation of an auricular mast cell tumour. An abdominal ultrasound and computed tomography were performed and an irregular, ill‐defined and partially mineralised lesion was observed around the caudal duodenal flexure without evidence of metastasis. The cytologic examination was highly suggestive of a granular cell tumour. Partial surgical ablation with histological and immunohistochemical examination of the lesion confirmed the diagnosis. According to our review of the literature, this is the first report documenting an abdominal granular cell tumour in a dog.

## Graphical abstract

An abdominal granular cell tumour with imaging, cytological and histopathological characteristic of malignancy was diagnosed in a 12‐year‐old mixed‐breed dog. This is the first report describing this type of tumour in the abdomen of the dog.

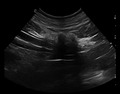

## 1 INTRODUCTION

Granular cell tumours, once called granular cell myoblastomas, are uncommon and usually benign lesions in both human and veterinary medicine. The oral cavity (notably the tongue) is the most common location in dogs, cats and people, but a wide range of other locations have been described (Higgins et al., 2017; Levitin et al., 2019; Mobarki et al., 2020; Patnaik, 1993). In dogs, documented locations for this type of tumour include the brain, meninges, spinal nerve root, heart, lung, eye, vocal cord and trachea (Levitin et al., 2019; Patnaik, 1993). According to our review of the literature, there is not a previous publication of an abdominal granular cell tumour in the dog.

## 2 CASE HISTORY

A 12‐year‐old neutered female mixed‐breed dog was presented for a follow‐up examination 8 months after ablation of an auricular grade II mast cell tumour. At presentation, the patient was bright, alert and responsive. All vital parameters were within normal limits. The owner did not report any gastrointestinal signs.

A complete blood cell count, serum biochemistry and abdominal ultrasound were performed as part of the oncologic examination. Mild normocytic, normochromic, non‐regenerative anaemia [RBC 5.84 × 10^6^/μl (5.5–8.5), Hb 13.7 g/dl (12–18), PCV 36% (37–55), reticulocytes 19,856 (0–60,000)] and moderate increase of alkaline phosphatase [1356.5 UI/L (20–156)] were detected. Otherwise, the blood work was unremarkable.

**FIGURE 1:**
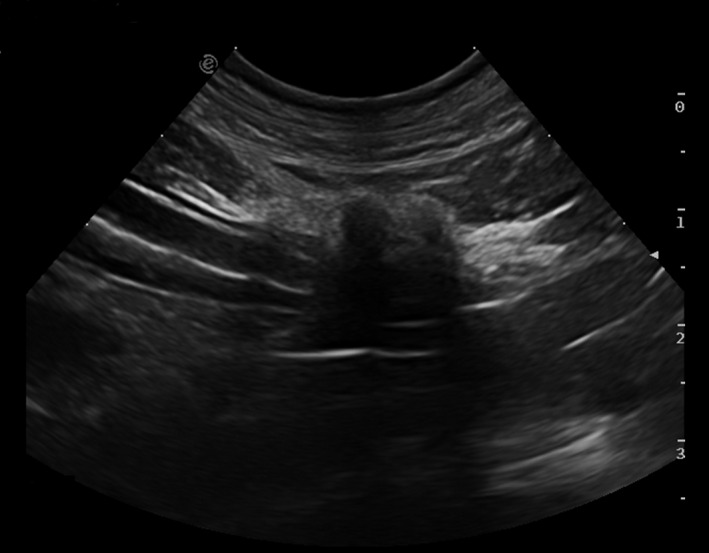
Sonographic image of the abdominal lesion affecting the mesentery and adjacent serosal layer of the caudal duodenum. The lesion at the mesenteric aspect of the intestinal loop is hyperechoic and ill‐defined with acoustic shadowing components

On the abdominal ultrasound an irregular and elongated lesion with ill‐defined margins was observed in the mesentery adjacent to the serosa of a small intestinal loop in the right caudal abdomen (Figure 1). The lesion measured 3.5 cm in length and 0.5 cm in thickness. It was markedly hyperechoic with several pin‐point hyperechoic foci that produced clean acoustic shadowing suggesting a mineral component. There was no other evidence of intestinal abnormalities. The adjacent mesenteric fat was moderately hyperechoic and heterogeneous. All the visualised abdominal lymph nodes were unremarkable. The most likely organ of origin was considered the mesentery with concurrent adhesion or infiltration of the adjacent intestinal serosa. An intestinal origin was considered less likely. The differentials included granulomatous tissue, fat necrosis or neoplasia (primary mesenteric or atypical metastasis from the previously reported mast cell tumour).

Ultrasound‐guided fine needle aspiration of the lesion was performed. On cytological examination a numerous and individually organised neoplastic cells were detected. They were large, round to slightly polygonal with eccentric round nuclei, reticular chromatin and occasional prominent nucleoli. The nucleus to cytoplasm ratio was low. There was abundant cytoplasm with distinct cytoplasmiizc borders and multiple variable shaped and sized granules. Mild anisocytosis and anisokaryosis were noted. Binucleated cells were frequent and multinucleated cells were occasionally encountered. No microorganisms were seen. The findings were highly suggestive of granular cell tumour. There was also mild mixed inflammation with neutrophilic and macrophagic predominance and evidence of previous haemorrhage.

**FIGURE 2:**
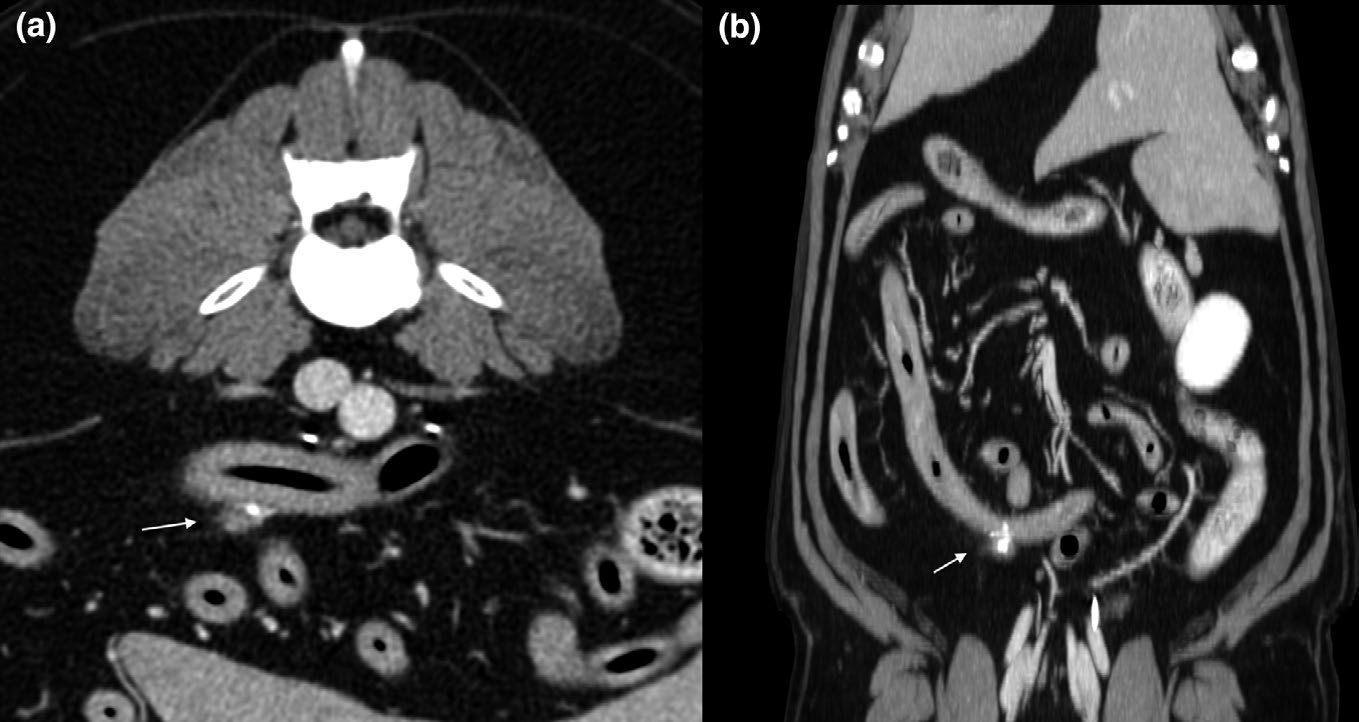
Computed tomographic study. (a) Transverse and (b) dorsal images of the lesion. It is adjacent to the distal duodenum. The granular cell tumour is hyperattenuating and ill‐defined with multiple pin‐point mineral foci

A thoracic and abdominal computed tomography (CT) was performed for staging and surgical purposes. An irregular and heterogeneous lesion with ill‐defined margins was observed adjacent to the serosa of the caudal duodenal flexure. The lesion was mainly soft tissue attenuation with multiple pint‐point foci of mineral attenuation and mild contrast enhancement (Figure 2). The surrounding fat was heterogeneous with subtle soft tissue striations. The affected intestinal loop was otherwise within normal limits as it was the rest of the gastrointestinal tract. The same possible origins and differentials were considered as in the ultrasound study. No other changes or lesions suggestive of metastatic process (e.g., lymphadenopathy) were found.

Laparoscopic approach was performed as the owners elected a less invasive approach. A multinodular, yellowish lesion was observed around the caudal duodenal flexure. The mass was firm and deeply attached to the intestinal wall and the surrounding mesenteric tissue, preventing its complete resection with the performed surgical approach. For the same reasons, full‐thickness biopsy or enterectomy were not performed.

**FIGURE 3:**
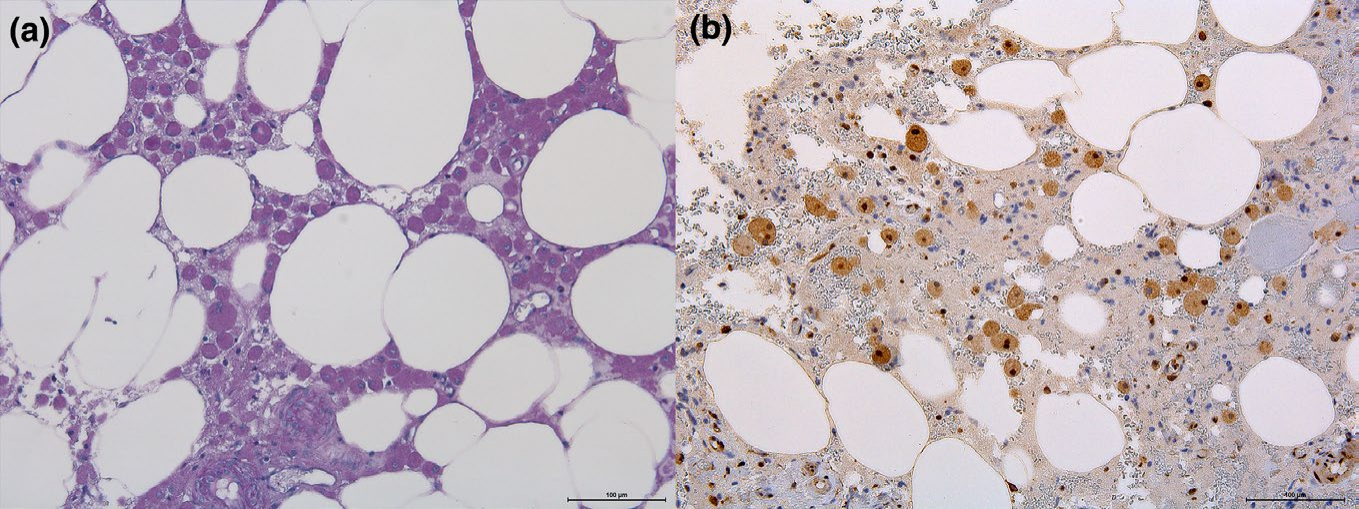
Round and large cell proliferation with eccentric nuclei; occasional multinucleated cells are present. Cytoplasmic granules are stained purple for periodic acid‐Schiff (PAS) (a) and show marked immune reaction against ubiquitin (b). Note the infiltrative pattern in mesenteric adipose tissue

The main histopathological finding was a neoplastic proliferation of the mentioned cells with a highly infiltrative pattern in adipose tissue. The cytoplasmic granules were periodic acid‐Schiff (PAS)‐positive, and immunohistochemistry revealed a strong reaction of the cells against ubiquitin (Figure 3). No positivity was observed under Luxol fast blue and S‐100 protein IHC. The diagnosis of granular cell tumour was confirmed based on the pathological, immunohistochemical and ultrastructural findings of the neoplastic tissue.

Six months after surgery the dog was alive and healthy without evidence of clinical signs.

## 3 DISCUSSION

According to our review of the literature, this is the first report describing an abdominal granular cell tumour in a dog. In this case, the tumour was in the mesentery and deeply attached to the serosal layer of the caudal duodenal flexure. Unfortunately, it was not possible to determine if the lesion originated from the mesentery or the intestinal serosa, as enterectomy or full‐thickness biopsy was not performed. In human medicine, up to 8%–11% of the granular cell tumours involve the gastrointestinal tract, most commonly the oesophagus, colon and stomach. Small intestine involvement is extremely rare (<1%), with only few cases of this tumour affecting the duodenum and ileum (Barakat et al., 2018; Nakachi et al., 2000; Radaelli & Minoli, 2009). Most gastrointestinal granular cell tumours in human medicine arise from the mucosal or submucosal layer (Mobarki et al., 2020; Nakachi et al., 2000). In the present case, the lesion was in close contact with the serosal layer and mesenteric adipose tissue, being the other intestinal layers normal in ultrasound and computed tomography. Based on these considerations, the mesentery is a more likely organ of origin compared to the intestine.

The histogenesis of the granular cell tumour remains controversial since its original description in 1926 (Patnaik, 1993). Initially, based on light microscopic features of the neoplastic cells, the tumour was thought to be of skeletal muscle in origin. Neurons, fibroblasts, histiocytes and myoepithelial cells are other potential origins that have been proposed in human medicine (Suzuki et al., 2015). Lately it is suspected that they arise from the neural crest, specifically the Schwann cells. Evidence that the canine and human granular cell tumours shares a similar origin has been recently published (Patnaik, 1993; Suzuki et al., 2015; Wilson, 2017). In our case, granular cells reacted markedly to PAS and ubiquitin, as it has been previously described (Higgins et al., 2017), but no positivity was observed under Luxol fast blue and S‐100 protein IHC. According to this results a Schwann cell origin was not confirmed. S‐100 negative granular cell tumours have been previously reported in veterinary medicine (Reifinger et al., 2020). A definitive explanation of this fact is beyond the aim of this study.

Some publications in human medicine have described findings suggestive of a previous foreign body in the histopathology of granular cell tumours (Meyer et al., 2010; Rocanti et al., 2013). In our case, the histological evaluation of the biopsy revealed (in addition to the granular cell population) some necrotic foci associated with reactive inflammatory cells. As no definitive foreign body was identified, those changes are considered secondary to the granular cell tumour.

Granular cell tumour are usually benign. Malignant transformation can happen, but this is rare in both human and veterinary medicine (Bouayyard et al., 2020; Higgins et al., 2017; Reifinger et al., 2020). In a recent human article, a 1%–2% of malignant conversion has been described (Bouayard et al., 2020). Few reports including malignant granular cell tumours have been published in veterinary patients (Patnaik, 1993). The present case has imaging, surgical and histopathological characteristics suggestive of local neoplastic infiltration, but no other indications of malignancy such as lymphadenopathy or evident metastatic disease were detected in the imaging studies.

Our patient presented for a follow‐up examination after ablation of an auricular mast cell tumour. To the authors’ knowledge, no correlation has been described between granular cell tumour and mast cell tumour in neither veterinary nor human literature. In absence of any evidence the authors assume that the two pathologies in this case are unrelated.

Ultrasound descriptions of granular cell tumours are lacking in veterinary medicine. Recently, ultrasound appearance of a celomic granular cell tumour has been described in a California kingsnake as a large mass, not associated with any organ, with cysts, and a wall of moderate echogenicity (Reifinger et al., 2020). In human literature, granular cell tumours affecting the gastrointestinal tract have been described as hypoechoic, homogenous, smooth‐edged solitary lesions that appear to originate from the submucosal layer (Barakat et al., 2018). Differently, in our case, the lesion was hyperechoic, heterogeneous and it appeared to arise from the mesentery or the intestinal serosal layer.

On MRI, intracranial granular cell tumours in veterinary medicine have been described as extensive and homogeneously enhancing extra‐axial mases (Anwer et al., 2013). Based on our review, CT description of this pathology in veterinary medicine is limited to an intracranial granular cell tumour in a dog. It is described as a well‐circumscribed, uniformly enhancing mass with well‐defined smooth margins (Higgins et al., 2017). On a deep review of human literature, nonspecific CT imaging characteristics were found with a range of different descriptions. No previous data are recorded in veterinary medicine except for the previously described study (Higgins et al., 2017), so it can be assumed that this variability on CT imaging characteristics could also be present in animals. In the present case, the granular cell tumour appeared as an ill‐defined, solid, heterogeneous and infiltrative lesion potentially arising from the mesentery or less likely from the serosal layer of the duodenum. Further studies are needed to better assess the CT characteristics of canine granular cell tumour.

In both ultrasound and CT exams, the imaging features suggested a mineral component of the neoplasm. However, no mineralisation was seen on histopathologic exam. A definitive explanation for this was not found but could be a consequence of only partial tumour resection. Interestingly, the presence of foci with acoustic shadowing has been also described in human breast granular cell tumour ultrasound (Carr‐Hoefer, 2003).

In conclusion, the abdomen (likely mesentery or the gastrointestinal tract) is a potential origin for canine granular cell tumour. Therefore, this tumour should be included in the differentials for a solitary, ill‐defined and irregular mass or lesion affecting the mesentery and/or the serosal layer of the gastrointestinal tract. Ultrasound and computed tomography are helpful for the diagnosis and presurgical planning. Histology is mandatory to confirm the diagnosis.

## CONFLICT OF INTEREST

No conflict of interest have been declared.

## AUTHOR CONTRIBUTION

**Alejandro Ororbia:** Conceptualization; Data curation; Investigation; Validation; Writing‐original draft; Writing‐review & editing. **Alba Sanz:** Data curation; Investigation; Validation; Writing‐original draft; Writing‐review & editing. **Rosa Novellas:** Data curation; Supervision; Validation. **Josep Pastor:** Data curation; Supervision; Validation. **Marti Pumarola:** Data curation; Formal analysis; Supervision; Validation; Writing‐original draft; Writing‐review & editing. **Laura Fresno:** Data curation; Resources; Supervision; Validation; Writing‐original draft. **Yvonne Espada:** Conceptualization; Data curation; Investigation; Supervision; Validation; Writing‐review & editing.

### PEER REVIEW

The peer review history for this article is available at https://publons.com/publon/10.1002/vms3.543.

## References

[vms3543-bib-0001] Anwer, C. C., Vernau, K. M., Higgins, R. J., Dickinson, P. J., Sturges, B. K., LeCouteur, R. A., Bentley, R. T., & Wisner, E. R. (2013). Magnetic resonance imaging features of intracranial granular cell tumors in six dogs. Journal of Veterinary Radiology and Ultrasound, 54(3), 271–277. 10.1111/vru.12027 23521525

[vms3543-bib-0002] Barakat, M., Abu Kar, A., Pourshahid, S., Ainechi, S., Lee, H. J., Othman, M., & Tadros, M. (2018). Gastrointestinal and biliary granular cell tumor: Diagnosis and management. Annals of Gastroenterology, 31(4), 439–447. 10.20524/aog.2018.0275 29991888 PMC6033765

[vms3543-bib-0003] Bouayyad, S., Ong, J., Bouayyad, H., & Woodun, H. (2020). Epiglottic granular cell tumour: A case report and literature review. Journal of Surgical Case Reports, 2, 1–4. 10.1093/jscr/rjaa009 PMC7031075 32099640

[vms3543-bib-0004] Carr‐Hoefer, C. (2003). Granular cell tumour: A benign mimicker of breast carcinoma. Journal of Diagnostic Medical Sonography, 19(2), 95–100.

[vms3543-bib-0005] Higgins, R. J., Bollen, A. W., Dickinson, P. J., & Meuten, D. J. (2017). Tumors of the nervous system. In D. J. Meuten (Ed.), Tumors in domestic animals (5th ed., pp. 870–872). John Wiley & Sons Inc.

[vms3543-bib-0006] Levitin, H. A., Foss, K. D., Hague, D. W., Connolly, S. L., Vieson, M., Wycislo, K. L., Lezmi, S., & Lovett, M. C. (2019). The utility of intraoperative impression smear cytology of intracranial granular cell tumours: Three cases. Veterinary Clinical Pathology, 48(2), 282–286. 31062410 10.1111/vcp.12732

[vms3543-bib-0007] Meyer, M. A., Becker, J. M., & Quinones, W. (2010). Endobronchial granular cell tumor: A case report. Journal of Radiology Case Report, 4(8), 29–35. 10.3941/jrcr.v4i8.474 PMC3303395 22470750

[vms3543-bib-0008] Mobarki, M., Dumollard, J. M., Dal Col, P., Camy, F., Peoc'h, M., & Karpathiou, G. (2020). Granular cell tumour a study of 42 cases and systemic review of the literature. Pathology – Research and Practice, 216(4). 10.1016/j.prp.2020.152865 32089415

[vms3543-bib-0009] Nakachi, A., Miyazato, H., Oshiro, T., Shimoji, H., Shiraishi, M., & Muto, Y. (2000). Granular cell tumor of the rectum: A case report and review of the literature. Journal of Gastroenterology, 35, 631–635. 10.1007/s005350070064 10955603

[vms3543-bib-0010] Patnaik, A. K. (1993). Histologic and immunohistochemical studies of granular cell tumors in seven dogs, three cats, one horse and one bird. Veterinary Pathology, 30, 176–185. 10.1177/030098589303000211 8470338

[vms3543-bib-0011] Radaelli, F., & Minoli, G. (2009). Granular cell tumors of the gastrointestinal tract questions and answers. Gastroenterology Hepatology, 5(11), 798–800. PMC2886372 37967389

[vms3543-bib-0012] Reifinger, M., Dinhopl, N., Gumpenberger, M., Konecny, M., & Cigler, P. (2020). Granular cell tumour in a California kingsnake (*Lampropeltis californiae*). Journal of Comparative Pathology, 175, 24–28. 10.1016/j.jcpa.2019.11.003 32138839

[vms3543-bib-0013] Roncati, L., Manco, G., Italia, S., Barbolini, G., Maiorana, A., & Rossi, A. (2013). Granular cell tumor of the appendix: A new case and review of the literature. SpringerPlus, 2(1), 10.1186/2193-1801-2-649 PMC3862858 24349953

[vms3543-bib-0014] Suzuki, S., Uchida, K., Harada, T., Nibe, K., Yamashita, M., Ono, K., & Nakayama, H. (2015). The origin and role of autophagy in the formation of cytoplasmic granules in canine lingual granular cell tumors. Veterinary Pathology, 52(3), 456–464. 10.1177/0300985814546051 25161210

[vms3543-bib-0015] Wilson, D. W. (2017). Tumors of the respiratory tract. In D. J. Meuten (Ed.), Tumors in domestic animals (5th ed., pp. 495–496). John Wiley & Sons Inc.

